# Sex-Based Differences in One-Year Outcomes After Mitral Valve Repair for Infective Endocarditis

**DOI:** 10.21470/1678-9741-2021-0333

**Published:** 2023-07-18

**Authors:** Zeinab Mohseni Afshar, Feridoun Sabzi, Maria Shirvani, Nahid Salehi, Nasim Nemati, Werya Kheradmand, Hadis Torbati, Mohammad Rouzbahani

**Affiliations:** 1 Behavioral Disease Research Center, Kermanshah University of Medical Sciences, Kermanshah, Iran; 2 Cardiovascular Research Center, Health Institute, Kermanshah University of Medical Sciences, Kermanshah, Iran; 3 Rajaie Cardiovascular Medical and Research Center, Iran University of Medical Science, Tehran, Iran

**Keywords:** Sex, Treatment Outcome, Endocarditis, Reoperation, Checklist

## Abstract

**Introduction:**

This study was aimed to evaluate the sex-based differences in baseline characteristics and one-year outcomes of men and women undergoing mitral valve repair for infective endocarditis.

**Methods:**

This cross-sectional study was performed at Imam Ali Hospital affiliated with the Kermanshah University of Medical Science. From March 21, 2014, to October 21, 2021, all patients who underwent mitral valve repair for infective endocarditis were enrolled in this study. Data were obtained using a checklist developed based on the study’s objectives. Independent samples *t*-tests, paired samples t-tests, and chi-squared test (or Fisher’s exact test) were used to assess the differences between subgroups.

**Results:**

Of 75 patients, 26 were women (34.7%) and 49 were men (65.3%). Women were more likely to have diabetes mellitus (20.4% *vs.* 57.7%, *P*=0.0001), hypertension (49% *vs.* 80.8%, *P*=0.007), and hypercholesterolemia (55.1% *vs.* 80.8%, *P*=0.027). Conversely, men were more likely to have a history of smoking (38.8% *vs.* 7.7%, *P*=0.004). After one year, women had significantly higher mortality (0% *vs.* 7.7%, *P*=0.049), major adverse cardiac and cerebrovascular events (51.0 *vs.* 76.9, *P*=0.029), mitral valve reoperation (8.1% *vs.* 34.6%, *P*=0.003), and treatment failure (30.6% *vs.* 61.5%, *P*=0.009) rates than men.

**Conclusion:**

Mortality, major adverse cardiac and cerebrovascular events, mitral valve reoperation, and treatment failure rates were higher in women than in men. The worse outcomes in women may be explained by their more adverse clinical risk profile.

## INTRODUCTION

Infective endocarditis (IE) is a known infection of the heart endothelium. It has an annual incidence of 3–10/100,000 and a mortality rate of 30%^[1,2]^. *Staphylococcus aureus* is now the most frequent cause of IE in most studies, being responsible for 26.6% of the cases, followed by viridans group streptococci, responsible for 18.7%, other streptococci, responsible for 17.5%, and enterococci, responsible for 10.5% of all cases. Overall, these organisms account for 80–90% of all endocarditis cases^[3]^.

Mitral valve repair (MVRep) has been continuously developed for decades and has been considered as the treatment of choice for degenerative mitral valve (MV) disease^[4]^. However, uncertainty remains on more challenging situations like IE^[5,6]^. Based on the recent guidelines^[7]^, early surgery should be applied for patients with active, left-sided IE to prevent clinical deterioration and embolization. In patients undergoing surgery for mitral IE, several studies and meta-analyses have proposed more favourable outcomes following valve repair, comparing to valve replacement^[7,8]^.

Men and women have major differences in the prevalence, response to treatment, and prognosis of cardiovascular disease (CVD). Historically, women have undergone less MV surgeries and will have worse clinical outcomes comparing with men. Chiu Wong et al.^[9]^ showed that women experienced higher in-hospital major adverse cardiac and cerebrovascular events (MACCE) compared with men after heart valve surgery. Vassiliev CM et al.^[10]^ found that women had higher operative mortality and lower long-term survival following MV surgery.

To the best of our knowledge, sex-based differences in one-year outcomes of MVRep for IE have not been suitably studied. Therefore, the current study was aimed to investigate sex-based differences in one-year outcomes after MVRep for IE.

## METHODS

### Study Population and Design

This cross-sectional study was conducted at the Imam Ali Hospital. This hospital provides advanced services in the cardiovascular field, with 280 active beds, annually admitting about two million patients. Therefore, the Imam Ali Hospital was selected for our study.

From March 21, 2014, to October 21, 2021, all the patients who underwent MVRep for IE were assessed for inclusion in this study. Patients aged ≥ 18 years presenting with a definite diagnosis of IE, according to modified Duke University criteria^[11]^, who underwent MVRep were selected (n=75). Patients were excluded from the study if they had congenital heart disease and had previously undergone MVRep.

MVRep was applied by using a downsized rigid or semi-rigid complete annuloplasty ring, with additional adjunctive approaches such as papillary muscle displacement, chordal shortening, chordal transferring, leaflet patch augmentation, and edge-to-edge repair in both groups, according to the surgeon’s discretion.

### Endpoints

Endpoints of interest comprised one-year all-cause death, a combination of MACCE (defined as the composite of death, stroke, MV reoperation, hospitalization for heart failure, myocardial infarction, cardiopulmonary resuscitation and cardiac arrest, an increase in New York Heart Association functional class ≥ I, and/ or cerebrovascular accident), treatment failure (defined as the composite of death, MV reoperation, or recurrence of IE), chest pain, symptom recurrence, and the degree of left ventricular (LV) reverse remodeling, which was assessed by the means of percent change in left ventricular end-systolic volume index (LVESVI) based on the transthoracic echocardiography done 12 months after MVRep.

### Instrument and Data Collection

Data were collected by a research assistant who was well trained to patient data entry and gathering. A valid checklist was used to obtain the data. The checklist was developed and verified by expert opinions compromising two cardiologists and a statistician. All completed checklists were checked and approved for errors by a general physician before final analysis. The collected data included demographic characteristics (*e.g.*, age), clinical history (*e.g.*, diabetes mellitus), medications (*e.g.*, statins), laboratory parameters (*e.g.*, creatine phosphokinase), echocardiographic data (*e.g.*, ejection fraction), and follow-up (*e.g.*, death). Standardized definitions of all variables (*e.g.*, clinical diagnoses) were used. Patients were assessed with a follow-up visit at the end of one postoperative year in the Imam Ali Hospital. We interviewed them to perform examinations and obtain patient history. Also, the patients underwent echocardiography at the end of the first postoperative year.

### Statistical Methods

Data analysis was performed using IBM Corp. Released 2015, IBM SPSS Statistics for Windows, Version 23.0, Armonk, NY: IBM Corp. Quantitative variables (*e.g.*, body mass index or age) were described using mean (standard deviation), and categorical data were expressed as frequencies (percentages). Differences between groups were assessed using independent *t*-tests for continuous and normally distributed variables and chi-squared test (or Fisher’s exact test) for categorical variables. Also, paired sample *t*-test was used to compare LVESVI in both women and men at one year after MVRep.

### Ethics

The Research Ethics Committee at Deputy of Research of the Kermanshah University of Medical Sciences approved the study protocol in March 2016 (IR.KUMS.REC.1398.702). Further, the participants had been given the participant information statement and had signed the written consent form. Individual personal information was kept confidential.

## RESULTS

Of 75 patients, 26 were women (34.7%) and 49 were men (65.3%). Baseline clinical characteristics of women and men are reported in Table 1. When men and women were compared, women were more likely to have diabetes mellitus (57.7% *vs.* 20.4%, *P*=0.0001), hypertension (80.8% *vs.* 49%, *P*=0.007), and hypercholesterolemia (80.8% *vs.* 55.1%, *P*=0.027). Conversely, men were more likely to have a history of smoking (38.8% *vs.* 7.7%, *P*=0.004). The mean level of fast blood sugar was significantly higher in women than men (118.59±39.93 *vs.* 166.46±78.61, *P*=0.001); the mean level of low-density lipoprotein was significantly higher in women than men (92.42±25.76 *vs.* 108.65±33.75, *P*=0.023); and the mean creatine kinase-myocardial band was also significantly higher in women than men (18.77±7.65 *vs.* 27.40±9.70, *P*=0.025).

**Table 1:** Baseline clinical characteristics in women and men undergoing MVRep for IE (n=75).

Characteristic	Men (n=49)¶	Women (n=26)¶	*P*-value
Age, years	59.80±8.20	59.53±10.25	0.902*
BMI, kg/m^2^	26.35±4.51	27.84±5.13	0.219*
Diabetes mellitus	10 (20.4)	15 (57.7)	0.001**
Hypertension	24 (49.0)	21 (80.8)	0.007**
History of smoking	19 (38.8)	2 (7.7)	0.004***
Hypercholesterolemia	27 (55.1)	21 (80.8)	0.027**
Hemoglobin (mg/dl)	12.98±1.89	12.91±2.24	0.887*
FBS (mg/dl)	118.59±39.93	166.46±78.61	0.001*
LDL (mg/dl)	92.42±25.76	108.65±33.75	0.023*
HDL (mg/dl)	37.55±7.07	37.38±9.89	0.933*
Triglycerides (mg/dl)	136.59±56.81	149.84±36.70	0.286*
CK-MB (units/L)	18.77±7.65	27.40±9.70	0.025*
BUN (mg/dl)	42.48±16.95	43.57±20.48	0.807*
Creatinine (mg/dl)	1.27±0.60	1.43±0.81	0.380*
Troponin I (ng/mL)	0.87±0.21	0.98±0.27	0.999*
ESR (mm/hour)	19.71±8.15	21.69±13.66	0.435*
Aspirin user	38 (77.5)	20 (76.9)	0.949**
ACE inhibitors user	18 (36.7)	11 (42.3)	0.637**
ARB user	21 (42.8)	12 (46.1)	0.784**
Beta blocker user	16 (32.6)	9 (34.6)	0.864**
Statin	39 (79.6)	22 (84.6)	0.595**

ACE=angiotensin-converting enzyme; ARB=angiotensin receptor blockers; BMI=body mass index; BUN=blood urea nitrogen;

CK-MB=creatine kinase-myocardial band; ESR=erythrocyte sedimentation rate; FBS=fast blood sugar; HDL=high-density lipoprotein; IE=infective endocarditis; LDL=low-density lipoprotein; MVRep=mitral valve repair

¶ Continuous variables expressed as mean ± standard deviation, otherwise n (%)

* *t*-test

** Chi-squared test

*** Fisher’s exact test

Baseline echocardiographic characteristics in women and men are shown in Table 2. When men and women were compared, men were more likely to have moderate/severe mitral regurgitation (MR) (69.4% *vs.* 50.0%, *P*=0.098). Compared with men, women had a smaller preoperative left ventricular end-systolic volume (LVESV) (133.54±53.10 *vs.* 96.10±41.58, *P*=0.001), LVESVI (66.75±25.23 *vs.* 53.15±22.81, *P*=0.021), and left ventricular end-diastolic volume (LVEDV) (211.45±64.61 *vs.* 172.15±47.75, *P*=0.004). Also, compared with men, women had a smaller postoperative LVESV (127.15±50.18 *vs.* 90.54±42.13, *P*=0.001), LVESVI (61.63±21.89 *vs.* 48.54±22.12, *P*=0.021), and LVEDV (205.12±66.78 *vs.* 166.45±44.11, *P*=0.012).

**Table 2:** Baseline echocardiographic characteristics and in women and men undergoing MVRep for IE (n=75).

Characteristic	Men (n=49)¶	Women (n=26)¶	*P*-value
**MVRep techniques**			0.933**
Ring undersizing + chordal transferring	9	6	
Ring undersizing + papillary muscle displacement	11	4	
Ring undersizing + edge-to-edge repair	10	6	
Ring undersizing + leaflet patch augmentation	8	5	
Ring undersizing + chordal shorting	11	5	
**Tricuspid regurgitation**			0.560**
Mild	33 (67.3)	19 (73.1)	
Moderate	14 (28.6)	7 (26.9)	
Severe	2 (4.1)	0 (0)	
**Mitral regurgitation**			0.098**
Mild	15 (30.6)	13 (50.0)	
Moderate/Severe	34 (69.4)	13 (50.0)	
Effective regurgitant orifice area of mitral valve, cm2	0.43±0.11	0.37±0.18	0.999*
**Preoperative**			
Left ventricular ejection fraction, %	38.46±6.78	36.53±10.74	0.344*
Left ventricular end-systolic volume, ml	133.54±53.10	96.10±41.58	0.001*
Left ventricular end-systolic volume index	66.75±25.23	53.15±22.81	0.021*
Left ventricular end-diastolic volume, ml	211.45±64.61	172.15±47.75	0.004*
Left ventricular end-diastolic volume index	111.22±29.68	99.55±35.81	0.162*
Systolic pulmonary artery pressure (mmHg)	38.40±12.45	35.15±11.42	0.445*
**Postoperative (1 year)**	**Men (n=49)**	**Women (n=24)**	***P*-value**
Left ventricular ejection fraction, %	37.55±8.29	36.45±8.90	0.607*
Left ventricular end-systolic volume, ml	127.15± 50.18	90.54 ± 42.13	0.001*
Left ventricular end-systolic volume index	61.63±21.89	48.54±22.12	0.021*
Left ventricular end-diastolic volume, ml	205.12±66.78	166.45±44.11	0.012*
Left ventricular end-diastolic volume index	107.78±25.91	94.13±38.42	0.125*
Systolic pulmonary artery pressure (mmHg)	37.51±8.40	37.29±8.84	0.917*

IE=infective endocarditis; MVRep=mitral valve repair

¶ Continuous variables expressed as mean ± standard deviation, otherwise n (%)

* *t*-test

** Chi-squared test

Clinical outcomes after one year for men and women are reported in Table 3. After one year, women had significantly higher mortality (0% *vs.* 7.7%, *P*=0.049), MACCE (51.0 *vs.* 76.9, *P*=0.029), MV reoperation (8.1% *vs.* 34.6%, *P*=0.003), and treatment failure (30.6% *vs.* 61.5%, *P*=0.009) rates than men.

**Table 3:** Clinical outcomes at one year in women and men after MVRep for IE (n=75).

Characteristic	Men (n=49)	Women (n=26)	*P*-value
All-cause mortality	0 (0)	2 (7.7)	0.049**
**Major adverse cardiac or cerebrovascular events** *	25 (51.0)	20 (76.9)	0.029**
Stroke	0 (0)	0 (0)	1**
MV reoperation	4 (8.1)	9 (34.6)	0.003**
Hospitalization for heart failure	2 (4.1)	3 (11.5)	0.217***
Hospitalization for MI	0 (0)	1 (3.8)	0.166**
Hospitalization for CPR and cardiac arrest	0 (0)	1 (3.8)	0.166***
Increase in NYHA functional class > I	23 (46.9)	11 (42.3)	0.701**
Cerebrovascular accident	0 (0)	0 (0)	1**
**Treatment failure** ¶	15 (30.6)	16 (61.5)	0.009**
Symptom recurrence	38 (77.5)	17 (65.4)	0.256**
Chest pain	16 (32.6)	11 (42.3)	0.407**

CPR=cardiopulmonary resuscitation; IE=infective endocarditis; MI=myocardial infarction; MV=mitral valve; MVRep=mitral valve repair; NYHA=New York Heart Association

* Defined as the composite of death, stroke, MV reoperation, hospitalization for heart failure, MI, CPR and cardiac arrest, an increase in NYHA functional class ≥ I, and/or cerebrovascular accident

** Chi-squared test

*** Fisher’s exact test

¶ Defined as the composite of death or MV reoperation

Likewise, LVESVI improved in both sexes after one year, however, differences between the preoperative and postoperative LVESVI in women (53.15±22.81 *vs.* 48.54±22.12, *P*=0.471) and in men (66.75±25.23 *vs.* 61.63±21.89, *P*=0.286) were not statistically significant.

Table 4 shows the treatment failure after one year classified according to repair methods.

**Table 4:** Treatment failure after one year classified according to repair methods.

Characteristic	Men (n=15)	Women (n=16)	*P*-value
Ring undersizing + chordal transferring	4 (26.7)	3 (18.8)	
Ring undersizing + papillary muscle displacement	3 (20.0)	5 (31.2)	0.48**
Ring undersizing + leaflet patch augmentation	4 (26.7)	4 (25.0)
Ring undersizing + edge-to-edge repair	4 (26.7)	4 (25.0)

¶Defined as the composite of death or mitral valve reoperation

** Chi-squared test

## DISCUSSION

To date, too limited data exist for sex-based differences in baseline characteristics and outcomes in patients with IE undergoing MVRep^[12]^. However, sex has been priorly demonstrated to affect the prognosis, response to treatment, and prevalence of CVDs^[13,14]^. Women with CVDs comprise an under detected, undertreated, and understudied patient population^[15]^. In this study, sex-based differences in baseline characteristics and one-year outcomes were compared between men and women undergoing MVRep for IE. To the best of our knowledge, this is the first study in the West of Iran to investigate differences in one-year outcomes by sex in IE patients undergoing MVRep.

The results of this study revealed that women illustrated a greater prevalence of comorbidities (*e.g.*, diabetes mellitus, hypertension, and hypercholesterolemia), while men were more likely to have a history of smoking. Smoking is known as a significant risk factor in men who suffers from CVD, whereas it is almost absent in women. In Iran, smoking is less common among women; therefore, women refuse smoking due to sociocultural factors. In line with our findings, Giustino et al.^[16]^ reported that despite being the same age at the time of MV surgery, women showed a higher prevalence of comorbidities (*e.g.*, diabetes mellitus, hypertension, and chronic kidney disease) than men in 2019. Vassileva et al.^[17]^ reported similar findings for patients undergoing MV surgery, showing women tended to be older and have more comorbidities. Conversely, Elbadawi et al.^[18]^ illustrated that compared with men, women undergoing MVRep had less chronic comorbidity. The sex-based difference in the clinical presentation that we and previous researchers have reported identifies the main issue for future improvement in care services for patients with valvular heart diseases. The less favorable profiles of women comparing with men may be associated with the physician’s referral bias. Women, especially in the Kurdish area, are mostly dependent on men financially, and therefore the decision to request treatment for women is made by men. Other issues, such as unawareness of comorbidities due to parturition, babysitting, housework and/or access to care, and/or a woman’s unwillingness to seek treatment, may also play a role.

Moreover, there were considerable echocardiographic differences between sexes. For instance, women had smaller LV volumes. Furthermore, change in LVESVI, as an alternative for LV inverse remodeling (LV remodeling measured with changes in LVESVI), almost similarly improved in men and women one year after MVRep. Consistently with our results, Giustino et al.^[16]^ reported that compared with men, women had smaller LV volumes as well as at two years after MV surgery, LVESVI improved in both women and men.

Women had significantly higher mortality, MACCE, MV reoperation, and treatment failure rates compared with men one year after MVRep. Giustino et al.^[16]^ reported that women had a significantly higher risk of mortality and MACCE compared with men after two years. Previous studies reported that among patients with primary severe MR, women had worse long-term outcomes than men (*e.g.*, mortality)^[19,20]^. Hirji et al.^[21]^ reported that women had significantly higher MV reoperation and operative mortality rates in 2020. Vassileva et al.^[17]^ illustrated similar findings for patients undergoing MV surgery in 2011, displaying women were more likely to die and had a longer mean length of stay compared with men. Conversely, Doshi et al.^[22]^ performed a study on patients undergoing transcatheter edge-to-edge MVRep, and they reported that there was no significant difference in the short-term outcomes by sex (*e.g.*, in-hospital mortality) in 2018. In contrast, Muñoz-Rivas et al.^[23]^, from Spain, showed that MACCE and in-hospital mortality rates were also significantly lower in women who underwent MV replacement in 2020. Elbadawi et al.^[18]^ demonstrated that there was no significant difference in in-hospital mortality and short-term outcomes for MVRep among women comparing with men in 2020.

The reasons for the worse outcomes in females after MVRep for IE stay unknown, are likely not totally ascribable to differences in responses to MVRep, and warrant further evaluations. Firstly, women had smaller LV values (LVESV indexed to body surface area) comparing with men, and changes in LVESVI over time have been considerably related to outcomes in patients. However, LVESVI improved in both sexes after one year, and differences between the mean changes in LVESVI in women and those in men were not significant. Secondly, in our study, the worse outcomes reported among women may be due to their significantly higher rates of chronic comorbidities at the time of MVRep. Indeed, a poorer risk profile increases the worse outcomes. In line with the results from the MV surveys^[19,20]^ and even other cardiac surgery studies^[24,25]^, the higher burden of chronic comorbidities at the time of surgery suggests a possible explanation for the observed adverse outcomes after MV surgery for IE in women when comparing with men.

### Limitations

Our study had several limitations. Firstly, the cross-sectional nature of the present study did not allow further evaluation of any apparent associations over time. Secondly, our data were obtained from a single center, therefore, our participants may not be representative of the whole patients who undergo MVRep for IE. Moreover, the sample size was small.

## CONCLUSION

Mortality, MACCE, MV reoperation, and treatment failure rates were higher in women than in their men counterparts. The worse outcomes in women after MVRep for IE may be explained by the more adverse clinical risk profile in women. Generally, tailoring the health care programs need to be improved, particularly for women, and their risk factors (including diabetes mellitus, hypertension, and hypercholesterolemia) should be timely modified to reduce the sex-based gaps in clinical outcomes.

## Abbreviations, Acronyms & Symbols

ACE: Angiotensin-converting enzyme
ARB: Angiotensin receptor blockers
BMI: Body mass index
BUN: blood urea nitrogen
CK-MB: Creatine kinase-myocardial band
CPR: Cardiopulmonary resuscitation
CVD: Cardiovascular disease
ESR: Erythrocyte sedimentation rate
FBS: Fast blood sugar
HDL: High-density lipoprotein
IE: Infective endocarditis
LDL: Low-density lipoprotein
LV: Left ventricular
LVEDV: Left ventricular end-diastolic volume
LVESV: Left ventricular end-systolic volume
LVESVI: Left ventricular end-systolic volume index
MACCE: Major adverse cardiac and cerebrovascular events
MI: Myocardial infarction
MR: Mitral regurgitation
MV: Mitral valve
MVRep: Mitral valve repair
NYHA: New York Heart Association
